# HIV Treatment as Prevention: Issues in Economic Evaluation

**DOI:** 10.1371/journal.pmed.1001263

**Published:** 2012-07-10

**Authors:** Till Bärnighausen, Joshua A. Salomon, Nalinee Sangrujee

**Affiliations:** 1 Department of Global Health and Population, Harvard School of Public Health, Boston, Massachusetts, United States of America; 2 Africa Centre for Health and Population Studies, University of KwaZulu-Natal, Mtubatuba, KwaZulu-Natal, South Africa; 3 Division of Global HIV/AIDS, Centers for Disease Control and Prevention, Atlanta, Georgia, United States of America; Duke University Medical Center, United States of America

## Abstract

Meyer-Rath and Over assert in another article in the July 2012 *PLoS Medicine* Collection, “Investigating the Impact of Treatment on New HIV Infections”, that economic evaluations of antiretroviral therapy (ART) in currently existing programs and in HIV treatment as prevention (TasP) programs should use cost functions that capture cost dependence on a number of factors, such as scale and scope of delivery, health states, ART regimens, health workers' experience, patients' time on treatment, and the distribution of delivery across public and private sectors. We argue that for particular evaluation purposes (e.g., to establish the social value of TasP) and from particular perspectives (e.g., national health policy makers) less detailed cost functions may be sufficient. We then extend the discussion of economic evaluation of TasP, describing why ART outcomes and costs assessed in currently existing programs are unlikely to be generalizable to TasP programs for several fundamental reasons. First, to achieve frequent, widespread HIV testing and high uptake of ART immediately following an HIV diagnosis, TasP programs will require components that are not present in current ART programs and whose costs are not included in current estimates. Second, the early initiation of ART under TasP will change not only patients' disease courses and treatment experiences—which can affect behaviors that determine clinical treatment success, such as ART adherence and retention—but also quality of life and economic outcomes for HIV-infected individuals. Third, the preventive effects of TasP are likely to alter the composition of the HIV-infected population over time, changing its biological and behavioral characteristics and leading to different costs and outcomes for ART.

## More versus Less Detailed Cost Functions

The results from the HTPN 052 trial reported in August 2011 demonstrated under the controlled conditions of a well-conducted clinical trial that early antiretroviral therapy (ART) can be highly effective in preventing transmission of HIV in stable heterosexual HIV-discordant couples [1]. Several experimental studies are currently underway or planned to investigate the effectiveness of HIV treatment as prevention (TasP) in general populations, including in HIV hyperendemic communities in sub-Saharan Africa [2],[3]. A few mathematical modeling studies have predicted the cost-effectiveness of TasP, using cost estimates derived from currently existing ART programs [4]–[7]. Meyer-Rath and Over review prior studies of ART costs, and discuss the cost assumptions used in economic evaluations of HIV treatment [8]. They find that economic evaluations of TasP have tended toward a simplified accounting for variation in ART costs across patients and settings, focusing on a limited set of factors such as regimen or disease stage. Meyer-Rath and Over argue that future economic evaluation should account for a range of other factors that may be significant determinants of ART costs, including scale and scope of delivery, health states, ART regimens, health workers' experience, patients' time on treatment, and the distribution of delivery across public and private sectors.

In making this argument, Meyer-Rath and Over distinguish between two categories of ART cost functions [8]: “cost accounting identities,” which generate estimates of total costs based on mathematical representations of the production process, and “flexible cost functions,” which generate estimates of total costs based on empirically derived relationships between costs and other factors, while treating the details of the production process as a “black box” (Text S1 of [8]). Meyer-Rath and Over find that that “[m]ost existing [ART] cost projections assume a single constant unit cost per patient-year, or per patient-year on a certain regimen,” while a few have allowed for variation of costs by disease stage but not by other factors [8]. Concerns with the level of detail in modeling the costs of TasP derive in part from the past focus on predictive, or *ex ante*, economic evaluations, which rely heavily on mathematical or statistical models to extrapolate from limited empirical observations (as opposed to *ex post* evaluations, which use direct observation of actual costs and benefits) [9].

It may indeed be ideal to capture the dependence of costs on many factors in economic evaluation of TasP—a task that could theoretically be achieved either by improving our understanding of the production process or through empirical examination of relationships between costs and other factors. However, the necessary data on the ART production process or on the relationship between ART costs and factors such as the scope of delivery or patients' time on treatment are currently largely lacking and may not become widely available for most settings in the near future, despite ongoing studies that will generate such data for a few settings. The absence of empirical data raises the question whether economic evaluation of TasP can be “good enough” without accounting for the dependence of ART costs on many of the factors that Meyer-Rath and Over argue convincingly could be determinants of ART costs.

The answer to this question will depend on both evaluation purpose and perspective. If the purpose is to decide whether or not to implement TasP, less detailed cost functions may be sufficient, because the result will be a yes/no answer indicating whether TasP produces a net benefit to society, or falls below some predetermined cost-effectiveness threshold. Such a result may be relatively robust to imprecision in the specification and estimation of costs. If, on the other hand, the purpose of the evaluation is to establish the most efficient approach to deliver TasP, given that it has been decided that it should be implemented, it will be crucial for the analysis to capture cost variations based on factors such as health worker–to–patient ratio, size and type of health care facility, and the level of integration of TasP programs into the general health care system.

The example Meyer-Rath and Over calculate in their article is a case in point. Based on theoretical considerations of economies of scale and empirical observation of scale effects in most industries, including in the delivery of HIV prevention services [10]–[12], they argue that it is unlikely that average costs would remain constant across scale of ART delivery. To demonstrate the potential impact of scale effects on costs, they adjust the estimates for implementing TasP in South Africa produced by Granich et al. [13] “for scale and a plausible pattern of distribution of patients into clinics” [8]. The result of this adjustment is an increase in total accumulated cost over 40 years from US$75 billion to US$106 billion. While this difference in cost estimates is large, given the dramatic effect of TasP estimated by Granich et al. [13], it may not alter the overall conclusion that TasP is a socially worthwhile intervention. In particular, if TasP could indeed eliminate HIV incidence, as Granich et al. [13] assert based on their modeling results, the economic case for TasP would likely be robust to large increases in the cost estimates. At the same time, cost increases of the magnitude calculated by Meyer-Rath and Over would be extremely important for the practical exercises of financial planning and budgeting for TasP.

Whether less detailed cost functions will suffice in a given situation is also affected by the evaluation perspective. For instance, from the perspective of national health policy makers, economic evaluation may not need to account for the relationship between ART costs and the sector of delivery because policy makers may not be concerned about patients who utilize ART in the private sector. Conversely, from the perspective of for-profit companies providing ART in workplace HIV treatment programs, only the private sector costs will be relevant. In such cases, cost functions that account for differences in public versus private sector costs are not required.

Meyer-Rath and Over start the important discussion of which factors to include in cost functions in economic evaluations of TasP. We argue that for particular evaluation purposes (e.g., to establish the social value of TasP) and from particular perspectives (e.g., national health policy makers) “undetailed” cost functions, which do not capture cost dependence on many factors, may be sufficient.

## ART and TasP Programs: Important Considerations for Economic Evaluation

A more fundamental issue in the economic evaluation of TasP is the fact that TasP programs will differ from existing ART programs in several important respects, which may substantially impact the costs, outcomes, and cost-effectiveness of TasP (for an overview, see Table 1).

**Table 1:** Overview of differences between existing ART programs and TasP.

Difference	Explanation
**Program components**	To achieve widespread and frequent HIV testing and high uptake of ART immediately following an HIV diagnosis, TasP strategies will likely require interventions that are not present in current ART programs.
**Disease experiences and treatment outcomes**	Disease experiences and treatment-relevant behaviors: patients who initiate ART early are less likely to experience recovery from the symptoms of the later stages of HIV disease. Lack of such experience may affect behaviors that are crucial for treatment outcomes, such as ART retention and adherence.
	Quality of life: early initiation may reduce quality of life (because it increases the duration of drug side effects and transforms people into patients several years earlier than under current ART guidelines) or improve quality of life (because it decreases the severity of drug side effects and avoids the psychologically distressing situation of having to wait for one's health status to deteriorate before being allowed to start ART).
	Economic productivity: early initiation may reduce the lifetime economic productivity of HIV-infected individuals (because it increases the total portion of lifetime spent utilizing ART) or improve productivity (because it avoids the negative economic consequences of deteriorating health preceding late ART initiation).
**Patient population**	In the long run, successful TasP could lead to changes in the composition of the people requiring ART, because the preventive effects of TasP may benefit some population subgroups at risk of HIV acquisition, but not others.

### Program Components and Costs

As Meyer-Rath and Over observe, estimates of ART costs in models predicting the cost-effectiveness of TasP are usually based on the mix of interventions in currently existing ART programs. However, TasP strategies will likely require interventions that are not usually present in existing ART programs [14]. The costs of these interventions need to be accounted for in economic evaluations of TasP. As data on CD4 count at ART initiation demonstrate [15],[16], most HIV-infected people in sub-Saharan Africa do not start treatment until they have reached advanced stages of HIV disease (despite a moderate but steady increase in median CD4 count at initiation over the past years [16]). The late ART initiation occurs despite national treatment guidelines in sub-Saharan Africa stipulating that all HIV-positive individuals be referred to CD4 count testing and clinical disease staging after a positive HIV test result [17].

To succeed in reducing HIV incidence in the general population, TasP programs require that very high proportions of all HIV-infected people in a community receive ART. To this end, TasP programs must ensure that most community members who have not been diagnosed with HIV frequently test for HIV and, if found to be HIV-infected, initiate treatment soon after diagnosis. The costs of interventions to achieve frequent, widespread HIV testing (e.g., through community mobilization and home-based testing) and high uptake of ART among people newly diagnosed with HIV (e.g., through counseling, improved transport, and financial incentives) must be included on the cost side of the economic evaluation of TasP [18].

In addition, some structures and processes that are in place in existing ART programs may need to be enhanced to achieve good TasP outcomes, e.g., community-based treatment supporters or mobile-phone short messages to ensure good ART retention and adherence [19]. The expenditures for such enhancements need to be accounted for in the economic evaluation of TasP. Of course, the specific components required for successful TasP will depend on the particular TasP intervention strategy—universal population-wide HIV testing and treatment will use different approaches, and incur different costs, than TasP strategies targeted at people at high risk of HIV transmission, such as HIV-infected individuals in HIV-discordant couples [2],[13],[20]. It will thus be crucial that TasP implementations both in trials and in routine settings are accompanied by rigorous empirical studies that measure the expenditures for all TasP components.

### Disease Experiences and Treatment Outcomes

A second issue in predicting the cost-effectiveness of TasP based on costs and outcomes observed in current ART programs is that TasP patients are initiated earlier on ART than patients in existing programs. While patients in sub-Saharan Africa currently initiate ART at a median CD4 cell count below 140 cells/µl [15] (which is substantially lower than the typical CD4 count eligibility thresholds of 200 cells/µl or 350 cells/µl), patients in successful TasP programs would initiate ART soon after first diagnosis of HIV infection (which ideally would occur soon after HIV infection). As a result of earlier ART initiation, patients' disease experiences and treatment outcomes are likely to be significantly altered.

#### Disease experiences and treatment-relevant behaviors

Patients who initiate ART early are unlikely to experience the symptoms of the later stages of HIV disease, as well as the subsequent recovery on treatment, which many patients enrolled in currently existing ART programs have experienced. The recovery on ART from weight loss, physical weakness, and the opportunistic infections of late-stage HIV disease may convince patients in current treatment programs that ART is indeed effective and, as a result, improve their long-term ART retention and adherence. Patients in successful TasP programs, on the other hand, will usually lack such experiences and may consequently be less motivated to adhere well to their clinical appointments and drug regimens. Rates of resistance development, mortality, and morbidity may thus be higher in TasP programs than in existing ART programs.

#### Quality of life

TasP is also likely to affect quality of life, but the direction and magnitude of the net effect over a patient's life course is unknown. TasP patients who initiate ART early will experience drug side effects of ART for a longer total duration than patients in currently existing programs. At the same time, side effects and toxicities may be less frequent or less severe in patients who initiate ART early [21],[22], and TasP patients who adhere well to their treatment regimens may be able to completely avoid some of the symptoms of more advanced stages of HIV disease that can substantially reduce quality of life [23]. It is further plausible that early ART initiation improves quality of life because it avoids the psychologically distressing situation of having to wait for one's health status to deteriorate before being allowed to start ART. Conversely, it is also plausible that early initiation reduces quality of life, because it transforms people with no obvious symptoms into patients, and burdens them with the responsibilities of chronic disease treatment, such as regular clinic visits and pill-taking, several years earlier than under current ART guidelines.

Importantly, the net effect of TasP on quality of life will depend on the counterfactual to which it is compared. Since economic evaluation is intended to inform the decision whether to implement TasP against the background of already existing policies, the best counterfactual will be ART provided at the current ART eligibility threshold in a country. In particular settings, such as the US, where individuals are currently already eligible at the highest CD4 count for which there is clear evidence of health benefit to the HIV-infected patient [24], TasP will be equivalent to initiating on ART a group of people who will not yet derive benefits for their own health from the treatment. In these situations, limiting the health effects of TasP will be limited to reduction of HIV transmission to others and quality of life changes. Empirical evaluations of TasP should thus always include quality of life assessments.

#### Economic productivity

In current ART programs, ART patients' economic situation commonly improves with time on treatment [25]–[27], and recent evidence from a population-based study shows that ART can lead to nearly full employment recovery among HIV patients in rural Southern Africa [28]. In the context of TasP, patients who adhere well to their treatment regimens will be unlikely to experience negative effects of HIV on economic productivity because they initiate ART many years before they would have suffered from significant HIV disease, had they not received ART. On the other hand, TasP patients will start incurring the time losses and transport costs of ART utilization several years earlier in their disease course than patients enrolled in currently existing ART programs. For economic evaluations that take the perspective of the society as a whole, which include patients' private expenditure and economic productivity in addition to the costs incurred by the public health care sector, ART effects on patient income must be incorporated in the analysis. As the direction and magnitude of TasP effects on economic outcomes over a patient's life course are currently unknown, these effects need to be established in empirical studies of TasP.

### Changes in the HIV-Infected Population

Above, we have argued that the same people would behave differently in TasP programs than they currently do in existing ART programs. But, over time, TasP will also change who the people living with HIV are. One important reason for this change arises because people who newly acquire HIV despite successful implementation of TasP in their communities are likely to differ in their biology or behavior from the people who have acquired HIV in the past and are currently receiving treatment. If TasP is indeed effective in averting onward transmission of HIV, the people who acquire HIV in the presence of TasP may have particularly weak immune-system functioning or engage in exceptionally high-risk sexual behavior with HIV-infected people who do not participate in TasP. Thus, under successful TasP strategies the population of HIV-infected individuals will not only be smaller (as the TasP effects reducing transmission will over time outweigh the effects on survival [29],[30]), but it will also possess different average characteristics, e.g., regarding biological susceptibility to HIV infection or sexual risk-taking. As average ART patient characteristics change following the introduction of TasP, it is likely that ART costs and outcomes will change as well, because some of the characteristics that determine the extent to which a person is protected from HIV acquisition by TasP will also affect ART success (such as immunological functioning or adherence behavior).

## Conclusion

Over the coming years, more detailed cost data are likely to become available, which will allow incorporating the cost determinants that Meyer-Rath and Over identify into predictive models of the economic value of TasP. While these data are not yet available, it is important to keep in mind that detail in representing the dependence of ART costs on a range of factors in economic evaluation is likely to matter far less for establishing whether TasP is beneficial for a population than for determining which models of TasP delivery allocate scarce resources optimally. For the former purpose, relatively simple cost functions may be sufficient.

Several more fundamental issues deserve consideration in setting up economic evaluations of TasP. First, to achieve frequent, widespread HIV testing and high uptake of ART immediately following an HIV diagnosis, TasP programs will likely require components that are not present in current ART programs and whose costs are thus not incorporated in current cost estimates (such as community mobilization and frequent HIV testing of all community members who have not been diagnosed with HIV). Second, the early initiation of ART under TasP will change not only patients' disease courses and treatment experiences (which can affect behaviors that determine clinical treatment success, such as ART adherence and retention), but also the quality of life and economic productivity of HIV-infected populations—changes in outcomes that need to be accounted for in economic evaluation. And, third, the preventive effects of TasP are likely to alter the composition of the HIV-infected population in the long run, changing its biological and behavioral characteristics and leading to ART costs and outcomes that are different from those observed for current ART patients.

Hence, while it is useful to predict the economic value of TasP using data from existing ART programs, such forecasts can only serve as an initial guide for health policy. Future studies accompanying ongoing and planned TasP trials and implementations need to comprehensively assess the costs of all TasP components, as well as TasP effects on a range of outcomes beyond HIV incidence and mortality, including quality of life and economic productivity.

**Key Points**

- Using antiretroviral treatment (ART) cost functions that capture a range of relationships between costs and other factors, as recommended by Meyer-Rath and Over in another article in this collection [8], will likely be important for economic evaluations aiming to determine which delivery model of HIV treatment as prevention (TasP) will allocate resources optimally, but it may not be necessary to establish whether TasP will increase social welfare.
- There are several problems inherent in predicting the cost-effectiveness of TasP based on outcomes and costs observed in currently existing ART programs, because TasP will (i) require components that are not present in existing programs, (ii) affect the disease courses and treatment experiences, quality of life, and economic productivity of people living with HIV, and (iii) change the composition of the HIV-infected population.
- To improve our capacity to estimate the social value of TasP in economic evaluation, empirical studies need to comprehensively assess the costs of all TasP components and measure not only the TasP effects on HIV incidence and mortality but also the impacts on quality of life and economic productivity.

## Abbreviations

ART: antiretroviral therapy
TasP: HIV treatment as prevention
